# Need for improving the health system preparedness for road traffic injuries in Nepal

**DOI:** 10.3126/nje.v8i3.23726

**Published:** 2018-09-30

**Authors:** Brijesh Sathian, Puspa Raj Pant, Edwin van Teijlingen, Indrajit Banerjee, Bedanta Roy

**Affiliations:** 1 Academic Research Associate, Clinical Research, Trauma and Vascular Surgery, Surgery Department, Hamad General Hospital, Doha, Qatar.; 2 Research Fellow, Nepal Injury Research Centre, University of the West of England, Bristol, UK.; 3 Professor, Centre for Midwifery, Maternal and Perinatal Health, Bournemouth University, Bournemouth, UK.; 4 Associate Professor, Department of Pharmacology, SSR Medical College, Belle Rive, Mauritius.; 5 Senior Lecturer, Department of Physiology, Quest International University Perak (QIUP), City Campus, Ipoh, Perak Darul Ridzuan, Malaysia.

The growth of motorised and mass transportation has improved the lives and lifestyles of many. This economic progress has major drawbacks including: increased air pollution, road traffic crashes/injuries (RTCs/RTIs). Some papers may refer to Road Traffic Accidents or RTAs, but experts recommend the more appropriate to use RTIs. In recent decades the proportion of RTI to all deaths has declined in high-income countries whereas it has risen in many low- and middle-income countries [1]. Causalities in low-income countries constitute 90% of all RTI deaths and disability-adjusted life years globally [1]. RTIs cause personal suffering but they are also an important public health and development problem [2]. RTIs also have an economic cost both to its victims and their families, but also to national economies [3].

RTI are the eighth leading cause of death globally, resulting in the loss of over 1.35 million lives and cause nonfatal injuries to as many as 50 million people annually [2]. RTIs are the main cause of death among people aged between 5 and 29 years [1, 2, 3], and over half of the people who die on the road globally are pedestrians, cyclists and motorcyclists [2]. Our paper outlines some of the key issues in Nepal and offers suggestions for improvement to make traveling safer in the long run.

## RTIs in Nepal

According to the Department of Transport almost 80% of the vehicles registered in Nepal are motorized two-wheelers (motorcycle/ scooter/moped). Understandably, motorcycle comprises the biggest proportion of road crashes throughout the country [1, 4-5]. In most low-income countries motorized two-wheeler occupants represent the highest proportion (73%) of deaths, followed by occupants of four wheelers (11%) and heavy vehicles (6%) [6-9]. The Global Burden of Diseases estimated that of the 6,788 road traffic deaths in 2017 where 53% were pedestrians, 19% motorcyclists and 20% were in motor vehicles [1], despite larger motor vehicles cause multiple casualties in a single event [10]. Whilst on the Prithvi Highway (from Chitwan to the capital Kathmandu) truck and tipper accidents are more common than those in other motorized vehicles [11]. Not a week goes by without a major accident some are so serious they make world news, such as the December 2018 crash in western Nepal when a bus carrying school students and teachers back from a botanical field trip plunged into a gorge, killing 23 people.

As elsewhere younger people in the productive age group are most likely to be injured or killed in RTIs. In Nepal (as for example in India) RTIs are most common in poor people those who are cyclists, pedestrians, and passengers on mini-buses [12]. Injury is associated with catastrophic spending especially for poorer people who are not able to go to hospital because of the high costs associated with hospital care [13].

Dangerous roads and drivers contribute to an unsafe transport system affecting the journeys to major tourist attractions. If foreign tourists perceive that travelling to their destinations can be risky, they may opt to go elsewhere on holiday. However, promoting Nepal as a tourist destination means more tourists using the roads which may be directly related to an increase in RTIs. A Spanish study has demonstrated how tourism can be associated with RTIs in touristic areas [14]. This can have a negative consequence on a country’s tourism sector. Estimates reveal that the economic burden of RTI in low and middle-income countries is between 1% and 1.5% of gross national product (GNP) and high-income countries 2% [8,15]. However, one study estimated Nepal to be among the top losers as it had lost the equivalent to US$ 500 million due to RTIs in 2004 [16].

## The need for RTI prevention in Nepal

There is clearly a lack of effective road safety interventions in Nepal. Data suggest that most crashes in Nepal occur from 8 AM to 8 PM [11], this is due to a higher volume of traffic between those hours. Adequate numbers of traffic police are needed to help prevent RTIs. These police officers need to be equipped with speed detection equipment, breathalyzers (to test for alcohol levels in drivers’ breath), body cameras and smartphones. Traffic rules should be enforced stricter with cameras at major road junctions as well as electronic traffic signals [17] and perhaps also in long-distance passenger and cargo vehicles. This Transport Research Laboratory Report sets out methods to enforce: speed limits, drink driving restrictions, traffic light violation, and seat belt legislation [17]. Although it was prepared for London’s road, the underlying general principles often apply to Nepal. Recently, the UK brought into effect tougher rules against use of mobile and booster seats in cars.

Many RTIs are preventable. Several countries have achieved reductions in the figure of crashes and injuries by creating and implementing stricter traffic laws, including (a) better policing of alcohol use on the road; (b) speed limits; (c) the use of seat-belts and child restraints; (d) crash helmets. Moreover, countries should be making vehicles more protective for occupants, pedestrians and cyclists; and formulating and implementing transport policies that encourage safety [18-20]. The Global Road Safety Partnership has been “creating and supporting multi-sector road safety partnerships that are engaged with front-line good practice road safety interventions in countries and communities throughout the world [19].” Furthermore, the United Nations has called for the Decade of Action against Road Injuries (2011-2020) [20]. The World Health Organization (WHO) has recently developed a road safety technical package Save LIVES which, based on the Safe System Approach, incorporates safe roads and surroundings, safe vehicles, safe road users and safe speed [3]. As outlined in the World Report on Road Traffic Injury Prevention, there is a range of interventions suitable for a low-income country [18].

## The way forward

Nepal Health Sector Strategy 2015-2020 aims to halve the road traffic-related deaths by 2020. Nepal will be nearer to this ambitious target only if enough attention is being paid to the actions outlined in the Nepal Health Sector Strategy Implementation Plan 2016-2021. The Government of Nepal can also seek help from International Non-Governmental Organisations (INGOs) and scientists to design, implement and evaluate the accident reducing intervention programmes. Since RTIs are the most common cause of injury and trauma in Nepal [21-22], the following issues should be addressed: speeding; drink-driving; improper use (or non-use) of helmets and seatbelts; overloading of all vehicles; vehicle fitness; road maintenance; and driver training.

In addition, the Government of Nepal should expand and promote community-based interventional programmes with the help of medical professionals and also need to setup paramedic services to provide urgent care. There are several hospitals based basic trauma care courses for medical professionals, but Nepal is not using this human resource effectively. Thus there should be more training on basic trauma care for health professionals to support road crash victims [23].

## References

[1] Global Burden of Disease Study 2017. Global Burden of Disease Study 2016 (GBD 2017) Results. Seattle, United States: Institute for Health Metrics and Evaluation (IHME), 2017 [cited 20 September 2018]. Available from https://vizhub.healthdata.org/gbd-compare/

[2] World Health Organization. Global status report on road safety 2018. World Health Organization, 2018 [cited 20 September 2018]. Available from https://www.who.int/violence_injury_prevention/road_safety_status/2018/en/

[3] World Health Organization. Save LIVES - A road safety technical package. Geneva: World Health Organization, 2017 [cited 20 September 2018]. Available from https://www.who.int/violence_injury_prevention/publications/road_traffic/save-lives-package/en/

[4] Huang L Adhikary KP Choulagai BP Wang N Poudyal AK Onta SR Road Traffic Accident and its Characteristics in Kathmandu Valley. Journal of the Nepal Medical Association, 2016; 55(1): issue 203. 27935914

[5] Nepal Traffic Police statistics. [cited April 2018]. Available from: https://www.nepalpolice.gov.np/images/statistic/webpage/index.html

[6] Jha N Goutam R Jagdish S Epidemiological study of Road traffic cases: A study from south India. IJCM. 2004; (1): 20-24.

[7] Jacobs G Aeron-Thomas A Astrop A Estimating global road fatalities. Transport Research Laboratory (TRL Report number 445): Berkshire, UK.

[8] Mondal P Kumar A Bhangale UD Tyagi D A silent tsunami on Indian Road: A comprehensive Analysis of epidemiological aspect of road traffic accidents. British Journal of Medicine & Medical Research, 2011; 1(1):14-23. 10.9734/BJMMR/2011/106

[9] Mishra B Sinha Mishra ND Sukhla S Sinha A Epidemiological study of road traffic accident cases from western Nepal. Indian Journal of Community Medicine, 2010; 35: 115-121. 20606934 10.4103/0970-0218.62568 PMC2888338

[10] Pearce T Maunder D Babu D Rwebangira T Bus Accidents in India, Nepal, Tanzania, and Zimbabwe. Transportation Research Record: Journal of the Transportation Research Board, 2000; 1726, 16-23. https://trrjournalonline.trb.org/doi/abs/10.3141/1726-03

[11] Choulagai B Ling H Sharma P Mishra SR Ahmed M Chand PB Epidemiology of Road Traffic Accidents in Nepal: Data Review and Qualitative Analysis. SM Journal of Public Health and Epidemiology 2015; 1(3):1014.

[12] Nantulya VM Reich MR The neglected epidemic: Road traffic injuries in developing countries. BMJ, 2002 324:1139–41. 10.1136/bmj.324.7346.1139 12003888 PMC1123095

[13] Saito E Gilmour S Rahman MM Gautam GS Shrestha PK Shibuya K Catastrophic household expenditure on health in Nepal: a cross-sectional survey. Bulletin of the World Health Organization 2014; 92:760-767. 10.2471/BLT.13.126615 25378730 PMC4208475

[14] Rosselló J. Saenz-de-Miera O. Road accidents and tourism: The case of the Balearic Islands (Spain). Accident Analysis & Prevention 2011;43(3): 675-683. 10.1016/j.aap.2010.10.011 PMid: 21376854

[15] Peden MM Krug E Mohan D Hyder A Norton R Mackay M Dora C Five-year WHO Strategy on Road Traffic Injury Prevention. Geneva: World Health Organization 2001 [http://www.who.int/violence_injury_prevention/media/en/156.pdf]

[16] Dalal K Lin Z Gifford M Svanström L Economics of Global Burden of Road Traffic Injuries and Their Relationship with Health System Variables. International Journal of Preventive Medicine. 2013;4(12):1442-1450. PMid: PMCid: 24498501 PMC3898451

[17] Elliott MA Broughton J How methods and levels of policing affect road casualty rates. Wokingham, Berks, UK: Transport Research Laboratory, 2005.

[18] World Health Organization. World Report on Road Traffic Injury Prevention. Geneva: WHO 2004:107-141

[19] Global Road Safety Partnership Drinking and Driving: A road safety manual for decision-makers and practitioners; 2007. Geneva. [cited 28 April 2018]. Available from: http://www.who.int/roadsafety/projects/manuals/alcohol/drinking_driving.pdf.

[20] United Nations Road Safety Collaboration. World unites to halt death and injury on the road. Geneva: WHO 2011 [cited 28 April 2018]. Available from: http://www.who.int/roadsafety/en

[21] Shrestha R Shrestha SK Kayastha SR A comparative study on epidemiology, spectrum and outcome analysis of physical trauma cases presenting to emergency department of Dhulikhel Hospital, Kathmandu University Hospital and its outreach centers in rural area. Kathmandu University Medical Journal 2013; 11:241–6. 10.3126/kumj.v11i3.12513 24442174

[22] Mishra SR Neupane D Bhandari PM Burgeoning burden of non-communicable diseases in Nepal: a scoping review. Global Health 2015; 11:32 10.1186/s12992-015-0119-7 26178459 PMC4504073

[23] Joshi SK Shrestha S A study of injuries and violence related articles in Nepal. Journal of the Nepal Medical Association 2009; 48:209–16. 10.31729/jnma.182 20795459

